# Defining the critical hurdles in cancer immunotherapy

**DOI:** 10.1186/1479-5876-9-214

**Published:** 2011-12-14

**Authors:** Bernard A Fox, Dolores J Schendel, Lisa H Butterfield, Steinar Aamdal, James P Allison, Paolo Antonio Ascierto, Michael B Atkins, Jirina Bartunkova, Lothar Bergmann, Neil Berinstein, Cristina C Bonorino, Ernest Borden, Jonathan L Bramson, Cedrik M Britten, Xuetao Cao, William E Carson, Alfred E Chang, Dainius Characiejus, A Raja Choudhury, George Coukos, Tanja de Gruijl, Robert O Dillman, Harry Dolstra, Glenn Dranoff, Lindy G Durrant, James H Finke, Jerome Galon, Jared A Gollob, Cécile Gouttefangeas, Fabio Grizzi, Michele Guida, Leif Håkansson, Kristen Hege, Ronald B Herberman, F Stephen Hodi, Axel Hoos, Christoph Huber, Patrick Hwu, Kohzoh Imai, Elizabeth M Jaffee, Sylvia Janetzki, Carl H June, Pawel Kalinski, Howard L Kaufman, Koji Kawakami, Yutaka Kawakami, Ulrich Keilholtz, Samir N Khleif, Rolf Kiessling, Beatrix Kotlan, Guido Kroemer, Rejean Lapointe, Hyam I Levitsky, Michael T Lotze, Cristina Maccalli, Michele Maio, Jens-Peter Marschner, Michael J Mastrangelo, Giuseppe Masucci, Ignacio Melero, Cornelius Melief, William J Murphy, Brad Nelson, Andrea Nicolini, Michael I Nishimura, Kunle Odunsi, Pamela S Ohashi, Jill O'Donnell-Tormey, Lloyd J Old, Christian Ottensmeier, Michael Papamichail, Giorgio Parmiani, Graham Pawelec, Enrico Proietti, Shukui Qin, Robert Rees, Antoni Ribas, Ruggero Ridolfi, Gerd Ritter, Licia Rivoltini, Pedro J Romero, Mohamed L Salem, Rik J Scheper, Barbara Seliger, Padmanee Sharma, Hiroshi Shiku, Harpreet Singh-Jasuja, Wenru Song, Per Thor Straten, Hideaki Tahara, Zhigang Tian, Sjoerd H van Der Burg, Paul von Hoegen, Ena Wang, Marij JP Welters, Hauke Winter, Tara Withington, Jedd D Wolchok, Weihua Xiao, Laurence Zitvogel, Heinz Zwierzina, Francesco M Marincola, Thomas F Gajewski, Jon M Wigginton, Mary L Disis

**Affiliations:** 1 Earle A. Chiles Research Institute, Robert W. Franz Research Center, Providence Cancer Center, Providence Portland Medical Center, Portland, OR, USA; 2 Department of Molecular Microbiology and Immunology and Knight Cancer Institute, Oregon Health and Science University, Portland, OR, USA; 3 Institute of Molecular Immunology and Clinical Cooperation Group "Immune Monitoring", Helmholtz Centre Munich, German Research Center for Environmental Health, Munich, Germany; 4 Departments of Medicine, Division of Hematology Oncology, University of Pittsburgh Cancer Institute, Pittsburgh, PA, USA; 5 Department of Surgery University of Pittsburgh Cancer Institute, Pittsburgh, PA, USA; 6 Department of Immunology, University of Pittsburgh Cancer Institute, Pittsburgh, PA, USA; 7 Department of Clinical Cancer Research, The Norwegian Radium Hospital, Oslo University Hospital, Oslo, Norway; 8 Memorial Sloan-Kettering Cancer Center, New York, NY, USA; 9 Howard Hughes Medical Institute, New York, NY, USA; 10 Medical Oncology and Innovative Therapy, Instituto Nazionale Tumori-Fondazione 'G. Pascale', Naples, Italy; 11 Beth Israel Deaconess Medical Center, Boston, MA, USA; 12 Harvard Medical School, Boston, MA, USA; 13 Institute of Immunology, FOCIS Center of Excellence, 2nd Medical School, Charles University, Prague, Czech Republic; 14 Goethe Universität Frankfurt Am Main,Medizinische Klinik II, Frankfurt Am Main, Germany; 15 IRX Therapeutics, New York, NY, USA; 16 Instituto Nacional para o Controle do Câncer, Instituto de Pesquisas Biomédicas, PUCRS Faculdade de Biociências, PUCRS, Porto Alegre RS Brazil; 17 Department of Translational Hematology and Oncology Research, Cleveland Clinic, Cleveland, OH, USA; 18 Department of Solid Tumor Oncology, Cleveland Clinic, Cleveland, OH, USA; 19 Department of Pathology, McMaster University, Hamilton, ON, Canada; 20 University Medical Center Mainz, III. Medical Department, Mainz, Germany; 21 Ribological GmbH, Mainz, Germany; 22 Chinese Academy of Medical Sciences, Beijing, China; 23 Institute of Immunology, National Key Laboratory of Medical Immunology, Second Military Medical University, Shanghai, China; 24 Department of Surgery, Ohio State University, Columbus, OH, USA; 25 Department of Surgery, University of Michigan Medical Center, Ann Arbor, MI; 26 Faculty of Medicine, Vilnius University, Vilnius, Lithuania; 27 University of Queensland, Brisbane, Australia; 28 Ovarian Cancer Research Center, University of Pennsylvania Medical Center, Philadelphia, A, USA; 29 Department of Medical Oncology, VU Medical Center, Cancer Center Amsterdam Amsterdam, The Netherlands; 30 Hoag Institute for Research and Education, Hoag Cancer Institute, Newport Beach, CA, USA; 31 Department of Laboratory Medicine, Nijmegen Centre for Molecular Life Sciences, Radboud University, Nijmegen Medical Centre, Nijmegen, The Netherlands; 32 Department of Medical Oncology, Dana-Farber Cancer Institute, Boston, MA, USA; 33 Department of Medicine, Brigham and Women's Hospital, Boston, MA, USA; 34 Harvard Medical School, Boston, MA, USA; 35 Academic Department of Clinical Oncology, University of Nottingham, Nottingham, UK; 36 Department of Immunology, Cleveland Clinic Foundation, Cleveland, OH, USA; 37 INSERM U872, Cordeliers Research Center, Paris, France; 38 Alnylam Pharmaceuticals, Inc., Cambridge, MA, USA; 39 Institute for Cell Biology, Department of Immunology, University of Tuebingen, Tuebingen, Germany; 40 Istituto Clinico Humanitas, IRCCS, Milan, Italy; 41 Oncology Department, Oncology Institute Bari, Italy; 42 University of Lund, Lund, Sweden; 43 CanImGuide Therapeutics AB, Hoellviken, Sweden; 44 University of California, San Francisco, CA and Celgene Corporation, San Francisco, CA, USA; 45 Intrexon Corporation, Germantown,MD, USA; 46 Department of Medicine, Harvard Medical School, Boston, MA, USA; 47 Dana-Farber Cancer Institute, Boston, MA, USA; 48 Bristol-Myers Squibb Company, Wallingford, Connecticut, USA; 49 Translational Oncology & Immunology Centre TRON at the Mainz University Medical Center, Mainz, Germany; 50 Department of Melanoma Medical Oncology, MD Anderson Cancer Center, Houston, TX, USA; 51 The Institute of Medical Science, The University of Tokyo, Tokyo, Japan; 52 Department of Oncology, the Sidney Kimmel Cancer Center at Johns Hopkins, Baltimore, MD, USA; 53 ZellNet Consulting, Inc., Fort Lee, NJ, USA; 54 Pathology and Laboratory Medicine, University of Pennsylvania, Philadelphia, PA, USA; 55 Rush University Cancer Center, Rush University Medical Center, Chicago, IL, USA; 56 School of Medicine and Public Health, Kyoto University, Kyoto, Japan; 57 Division of Cellular Signaling, Institute for Advanced Medical Research, Keio University School of Medicine, Tokyo, Japan; 58 Dept. of Hematology and Medical Oncology, Charité Comprehensive Cancer Center, Berlin, Germany; 59 Cancer Vaccine Section, NCI, NIH, Bethesda, MD, USA; 60 Department of Oncology - Pathology, Cancer Center Karolinska, Karolinska Institute, Karolinska University Hospital, Stockholm, Sweden; 61 Department of Molecular Immunology and Toxicology, Center of Surgical and Molecular Tumor pathology, National Institute of Oncology, Budapest, Hungary; 62 INSERM, U848, Institut Gustave Roussy, Villejuif, France; 63 Research Center, University Hospital, Université de Montréal (CRCHUM), Montréal, Québec, Canada; 64 Institut du Cancer de, Montréal, Montréal, Québec, Canada; 65 School of Medicine, Oncology Center, Johns Hopkins University, Baltimore, MD, USA; 66 Department of Molecular Oncology, Foundation San Raffaele Scientific Institute, Milan, Italy; 67 Medical Oncology and Immunotherapy, Department of Oncology, University, Hospital of Siena, Istituto Toscano Tumori, Siena, Italy; 68 Merck KGaA, Darmstadt, Germany; 69 Department of Medical Oncology, Thomas Jefferson University, Philadelphia, PA, USA; 70 Department of Oncology-Pathology, Karolinska Institute, Stockholm, Sweden; 71 Department of Immunology, CIMA, CUN and Medical School University of Navarra, Pamplona, Spain; 72 Deptartment of Immunohematology and Blood Transfusion, Leiden University Medical Centre, Leiden, the Netherlands; 73 University of California-Davis Medical Center, Sacramento, CA, USA; 74 Deeley Research Centre, BC Cancer Agency, Victoria, BC, Canada; 75 Department of Internal Medicine, University of Pisa, Santa Chiara Hospital, Pisa, Italy; 76 Oncology Institute, Loyola University Medical Center, Cardinal Bernardin Cancer Center, Maywood, IL, USA; 77 Department of Gynecologic Oncology, Tumor Immunology and Immunotherapy Program, Roswell Park Cancer Institute, Buffalo, NY, USA; 78 Ontario Cancer Institute/University Health Network, Toronto, ON, Canada; 79 Cancer Research Institute, New York, NY, USA; 80 Ludwig Institute for Cancer Research, New York, NY, USA; 81 Experimental Cancer Medicine Centre, University of Southampton Faculty of Medicine, Southampton, UK; 82 Cancer Immunology and Immunotherapy Center, Saint Savas Cancer Hospital, Athens, Greece; 83 Unit of Immuno-Biotherapy of Melanoma and Solid Tumors, San Raffaele Scientific Institute, Milan, Italy; 84 Center for Medical Research, University of Tuebingen, Tuebingen, Germany; 85 Istituto Superiore di Sanita', Rome, Italy; 86 Chinese PLA Cancer Center, Nanjing, China; 87 The John van Geest Cancer Research Centre, School of Science and Technology, Nottingham Trent University, Nottingham, UK; 88 Department of Medicine, Jonsson Comprehensive Cancer Center, UCLA, Los Angeles, California, USA; 89 Immunoterapia e Terapia Cellulare Somatica, Istituto Scientifico Romagnolo per lo Studio e la Cura dei Tumori (I.R.S.T.), Meldola (FC), Italy; 90 Unit of Immunotherapy of Human Tumors, IRCCS Foundation, Istituto Nazionale Tumori, Milan, Italy; 91 Division of Clinical Onco-Immunology, Ludwig Center for Cancer Research of the University of Lausanne, Epalinges, Switzerland; 92 Immunology and Biotechnology Unit, Department of Zoology, Faculty of Science, Tanta University, Egypt; 93 Dept. of Pathology, VU University Medical Center, Amsterdam, The Netherlands; 94 Institute of Medical Immunology, Halle, Germany; 95 MD Anderson Cancer Center, Houston, TX, USA; 96 Department of Cancer Vaccine, Mie University Graduate School of Medicine, Mie, Japan; 97 Department of Immuno-gene Therapy, Mie University Graduate School of Medicine, Mie, Japan; 98 Immatics Biotechnologies GmbH, Tübingen, Germany; 99 Millennium: The Takeda Oncology Company, Cambridge, MA, USA; 100 Center for Cancer Immune Therapy (CCIT), Department of Hematology, Herlev Hospital, Herlev, Denmark; 101 Department of Surgery and Bioengineering, Advanced Clinical Research Center, Institute of Medical Science, The University of Tokyo, Tokyo, Japan; 102 Institute of Immunology, School of Life Sciences, University of Science & Technology of China, Hefei, China; 103 Institute of Immunopharmacology & Immunotherapy, School of Pharmaceutical Sciences, Shandong University, Jinan, China; 104 Experimental Cancer Immunology and Therapy, Department of Clinical Oncology, Leiden University Medical Center, Leiden, Netherlands; 105 Euraccine Consulting Group, Brussels, Belgium; 106 Infectious Disease and Immunogenetics Section (IDIS), Department of Transfusion Medicine, Clinical Center, NIH, Bethesda, MD, USA; 107 Center for Human Immunology (CHI), NIH, Bethesda, MD, USA; 108 Experimental Cancer Immunology and Therapy, Department of Clinical Oncology (K1-P), Leiden University Medical Center, Leiden, The Netherlands; 109 Department of Surgery, Klinikum Grosshadern, Ludwig Maximilians University, Munich, Germany; 110 Society for Immunotherapy of Cancer, Milwaukee, WI, USA; 111 Institute of Immunology, School of Life Science, University of Science and Technology of China, Hefei, China; 112 Institut Gustave Roussy, Center of Clinical Investigations CICBT507, Villejuif, France; 113 Department Haematology and Oncology Innsbruck Medical University, Innsbruck, Austria; 114 University of Chicago Medical Center, Chicago, IL, USA; 115 Discovery Medicine-Oncology, Bristol-Myers Squibb Company, Princeton, New Jersey, USA; 116 Tumor Vaccine Group, Center for Translational Medicine in Women's Health, University of Washington, Seattle, WA, USA

## Abstract

Scientific discoveries that provide strong evidence of antitumor effects in preclinical models often encounter significant delays before being tested in patients with cancer. While some of these delays have a scientific basis, others do not. We need to do better. Innovative strategies need to move into early stage clinical trials as quickly as it is safe, and if successful, these therapies should efficiently obtain regulatory approval and widespread clinical application. In late 2009 and 2010 the Society for Immunotherapy of Cancer (SITC), convened an "Immunotherapy Summit" with representatives from immunotherapy organizations representing Europe, Japan, China and North America to discuss collaborations to improve development and delivery of cancer immunotherapy. One of the concepts raised by SITC and defined as critical by all parties was the need to identify hurdles that impede effective translation of cancer immunotherapy. With consensus on these hurdles, international working groups could be developed to make recommendations vetted by the participating organizations. These recommendations could then be considered by regulatory bodies, governmental and private funding agencies, pharmaceutical companies and academic institutions to facilitate changes necessary to accelerate clinical translation of novel immune-based cancer therapies. The critical hurdles identified by representatives of the collaborating organizations, now organized as the World Immunotherapy Council, are presented and discussed in this report. Some of the identified hurdles impede all investigators; others hinder investigators only in certain regions or institutions or are more relevant to specific types of immunotherapy or first-in-humans studies. Each of these hurdles can significantly delay clinical translation of promising advances in immunotherapy yet if overcome, have the potential to improve outcomes of patients with cancer.

## Introduction

Globally, cancer claimed an estimated 7.6 million lives in 2008 and is on pace to double that number by 2030 [1]. The impact of this disease on humanity is difficult to measure. The Milken Institute estimates that in the United States (US) alone, a 1% reduction in cancer mortality has an economic value of $500 billion [2]. Currently the National Cancer Institute (NCI), National Institutes of Health (NIH), foundations, governments, biotechnology and pharmaceutical companies around the world are investing substantially in research to conquer this disease. Over the past decade, discoveries in basic cancer research related to this investment have provided an enormous number of insights, reagents, drugs and clinical protocols with potential to significantly improve cancer outcomes. Nowhere is this potential more striking and relevant to a wide spectrum of human cancers than in research on cancer immunotherapy, which has the capacity to provide durable clinical responses in even the most challenging cancers. Nonetheless, the translation of these discoveries from the "bench to the bedside" has been painfully slow.

**Figure 1:**
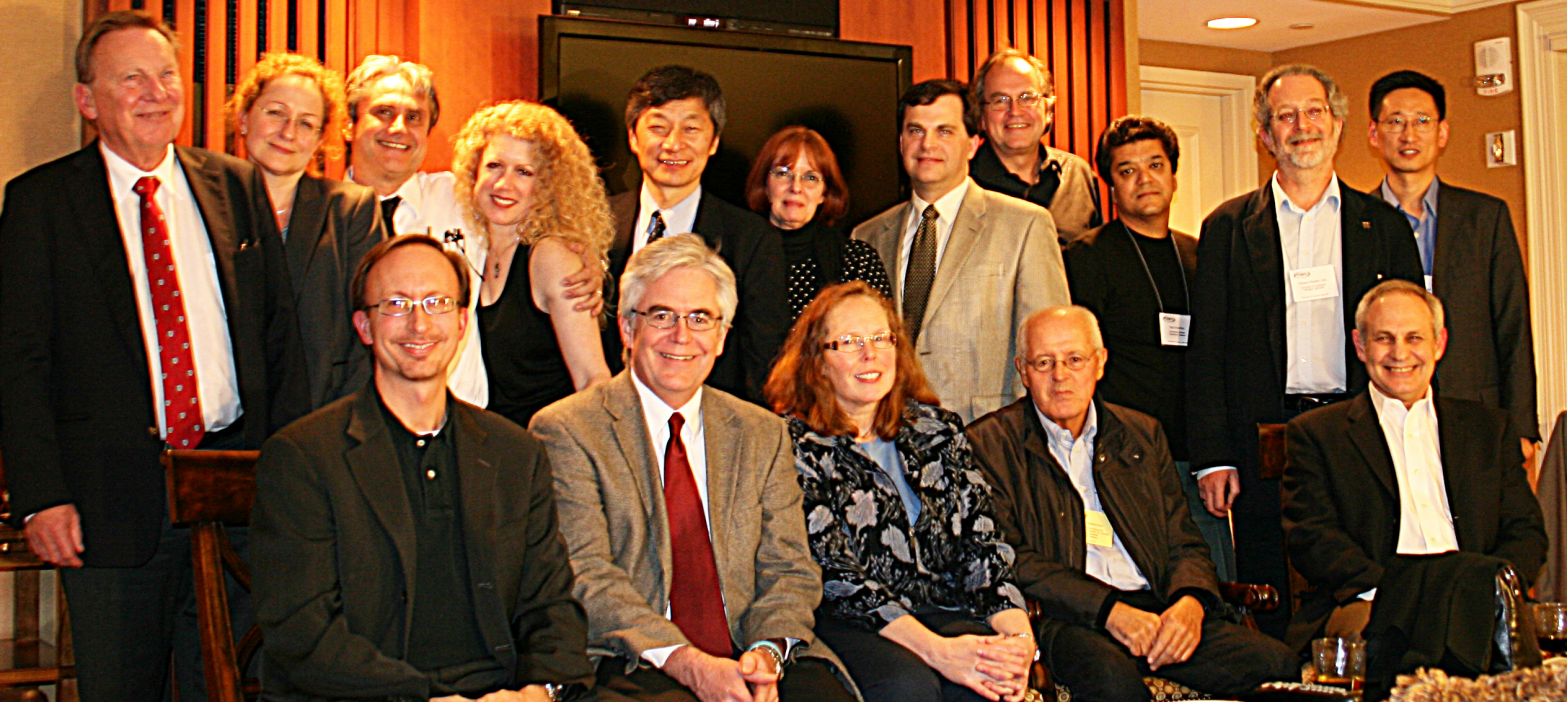
**2009 Immunotherapy Summit at SITC creating the working group, National Harbor, MD, USA**. Back row: Leif Haakason, Sylvia Janetski, Franco Marincola, Lisa Butterfield, Hideaki Tahara, Dolores Schendel, F Stephen Hodi, Heinz Zwierzina, A. Raja Choudhury, Graham Pawlec, Wenru Song. Front row: Tom Gajewski, Bernard A. Fox, Mary Disis, Michael Papamichail, Michael B. Atkins

**Figure 2:**
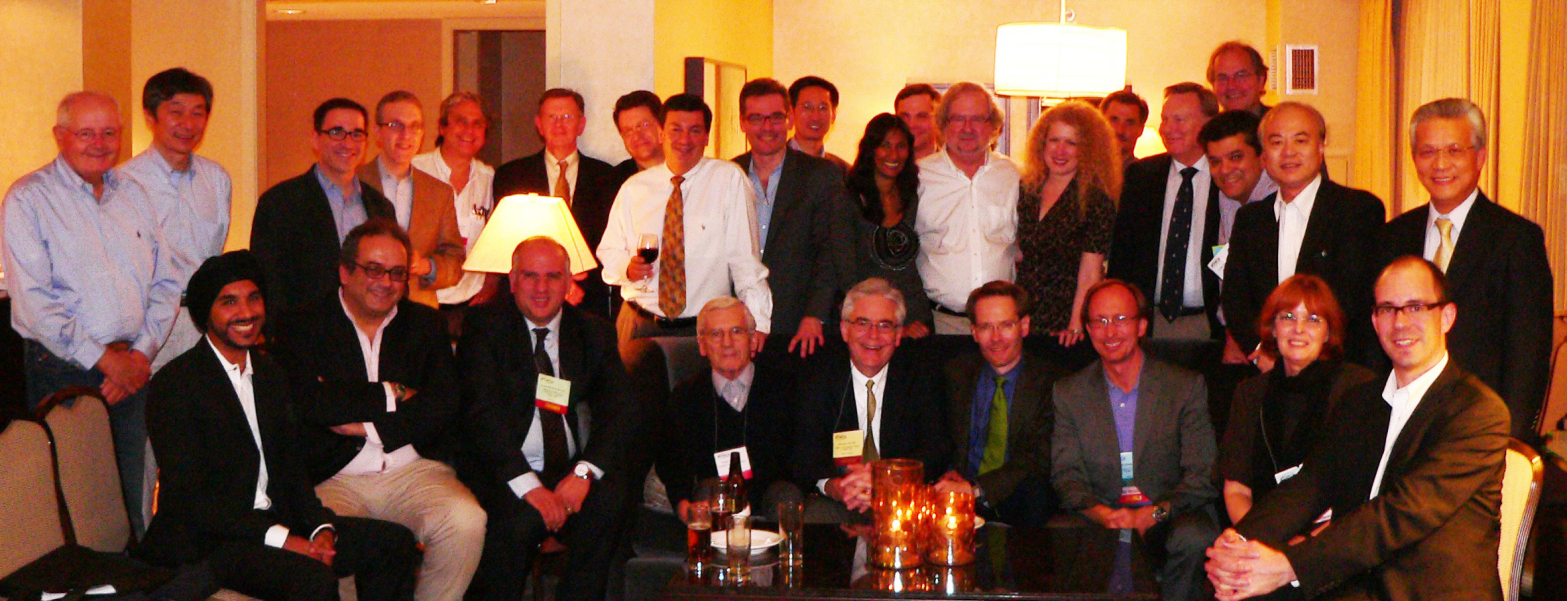
**2010 Immunotherapy Summit at SITC, Capital Hill, Washington DC, USA**. Back row: Michael Papamichail, Hideaki Tahara, Howard Kaufman, Jedd Wolchok, Franco Marincola, James Finke, Rejean Lapointe, Hyam I. Levitsky, George Coukos, Wenru Song, Padmanee Sharma, F Stephen Hodi, Jim Allison, Lisa Butterfield, William Murphy, Leif Haakson, A. Raja Choudhary, Heinz Zwierzina, Yutaka Kawakami, Kohzoh Imai. Front row: Harpreet Singh-Jasuja, Michele Maio, Paolo Ascierto, Giorgio Parmiani, Bernard A. Fox, Axel Hoos, Tom Gajewski, Dolores Schendel, Cedrik Britten.

In an effort to accelerate translation of new developments in basic immunology into patients with cancer, representatives from eight immunotherapy organizations representing Europe, Japan, China and North America (Figure 1) convened an "Immunotherapy Summit" at the 24^th ^Annual Meeting of the International Society for Biological Therapy of Cancer (iSBTc; now the Society for Immunotherapy of Cancer, SITC). One of the concepts raised by SITC and defined as critical by all parties was the need to identify hurdles that impede effective translation of cancer immunotherapy. Subsequently, ten organizations (Figure 2) met again in late 2010 at the 25^th ^Annual Meeting of SITC to discuss next steps and to commit to regular conference calls. While this is an important first step, identification of these hurdles is just the beginning. The development of collaborative, international working groups to identify solutions and help remove these hurdles could increase the speed at which novel, effective immunotherapy strategies reach patients with cancer. That is the goal.

**Figure 3:**
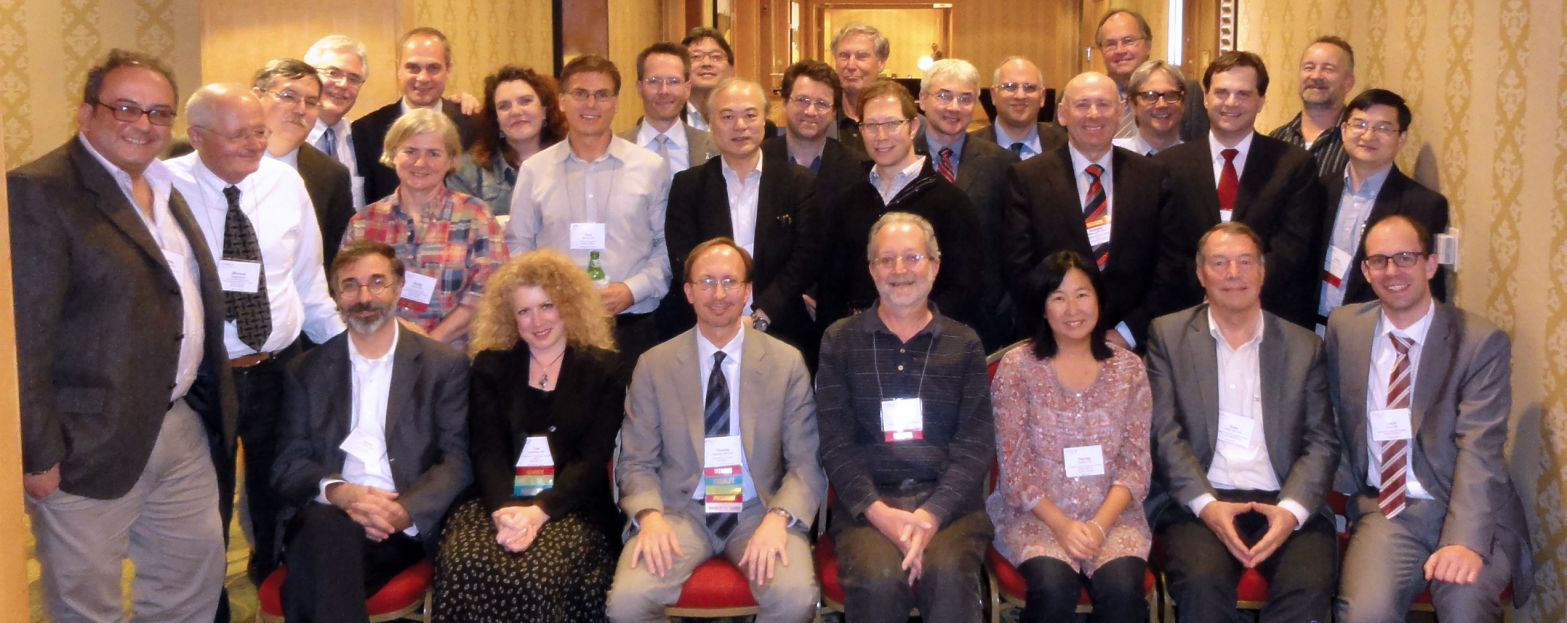
**2011 Immunotherapy Summit at SITC, North Bethesda, MD, USA**. Back row: Michele Maio, Michael Papamichail, Michael Nishimura, Bernard A. Fox, Andrea Nicolini, Jens-Peter Marschner, Tanja de Gruijl, Brad Nelson, Axel Hoos, Tetsuro Sasada, Yutaka Kawakami, Rejean Lapointe, Christoph Huber, Jonathan L. Bramson, Pawel Kalinski, Paolo Ascierto, Giuseppe Masucci, Heinz Zwierzina, Franco Marincola, F Stephen Hodi, Per Thor Straten, Jianda Yuan, Front row: Samir Khleif, Lisa Butterfield, Tom Gajewski, Graham Pawlec, Pam Ohashi, Cornelius Melief, Cedrik Britten.

The hurdles identified by representatives of the (now fifteen) collaborating organizations (Figure 3) can be grouped into nine general themes (Table 1). In some instances an identified hurdle is substantially interconnected with another hurdle or set of hurdles. For example, the lack of validated biomarkers further complicates the design and evaluation of clinical trials that combine immunotherapeutic agents. Thus efforts to address the identified hurdles to the translation of cancer immunotherapy must be through a coordinated, integrated, multidisciplinary and international approach.

**Table 1:** Critical Hurdles in Cancer Immunotherapy Identified by SITC and Collaborating Associations

1.	Limitations of current animal models to predict efficacy of cancer immunotherapy strategies in humans
2.	Prolonged time to obtain approval to initiate clinical trials
3.	Complexity of cancer, tumor heterogeneity and immune escape
4.	Limited availability of reagents for combination immunotherapy studies
5.	Limited funds available to translate science into patients
6.	Lack of definitive biomarker(s) for assessment of clinical efficacy of cancer immunotherapies
7.	Conventional clinical response criteria do not take into consideration differences between response patterns to cytotoxic agents and immunotherapies
8.	Paucity of teams of scientists and clinicians dedicated to translational research in cancer immunotherapy
9.	Insufficient exchange of information critical to advancing the field

What is Cancer Immunotherapy? Cancer immunotherapy is the original targeted therapy and includes any strategy that utilizes the anticancer immune response or components of the immune system, as cancer treatment. Seventeen immunotherapy products have received FDA approval in the past quarter century [3]. These include non-specific stimulators, cytokines, monoclonal antibodies, radiolabelled antibodies, immunotoxins, and cell-based therapy (reviewed in [3]). Further, the recent observations that immune response, characterized by immunohistochemistry, has better prognostic power than standard staging systems underscores the importance the endogenous immune response plays in patient outcomes and the potential impact boosting this immune response has for increasing survival [4,5]. These findings may help to recast the current classification, and to identify the high-risk patients who would benefit the most from adjuvant therapy.

### 1. Limitations of Preclinical Animal Models

While preclinical animal models have provided the basis for our understanding of immune function and significant insights into the mechanisms that regulate therapeutic efficacy of immunotherapy, the current models have not been consistent predictors for the efficacy of cancer immunotherapy strategies that enter the clinic. One reason for this disconnect may be that small, transplantable tumors, established for 3-5 days in an animal model, fail to recapitulate the complex, integrated pathophysiological setting, in which patients can have a large tumor burden that they have lived with for months to years. Models that utilize advanced or spontaneous tumors may begin to address this shortcoming. Another limitation is the inherent "immunogenicity" of the tumor model used. Experiments with tumors expressing xenogeneic proteins are frequently coupled with transgenic T cells to address basic questions about T cell trafficking, cytokine profiles and clonal expansion, in addition to many other scientific questions relevant to understanding the immunological response to tumors. However, given the foreign nature of the xenogeneic protein and the ease with which an immune response can be generated against these targets in wild type (WT) mice, these tumors are considered inadequate for modeling the human immune response to immunotherapy strategies. In other cases, the use of transplantable tumors without xenogeneic protein constructs may be useful. Further, many of the frequently used tumor cell lines were generated 20 - 40 years ago; given the genetic drift possible in 100 generations, the inbred mice may exhibit substantial histocompatibility differences that can result in these tumors being more immunogenic today than when they were originally developed, potentially limiting their usefulness as models of human disease. Another limitation is that the vast majority of studies are done in genetically identical inbred animals that do not represent the genetic diversity found in humans or in young mice, lacking the impact of aging on the immune system [6]. Some therapeutic interventions are tested in human xenograft models in immune-deficient mice, in which effects on and by the immune system are not addressed [7]. Human xenograft models in which human immune cells are also transferred are a potential improvement [8], although the reality of a fully functional human immune system in a mouse is still far away. Recently, severely immunodeficient mouse strains have been developed such as NOD.Cg-*Prkdc*^*scid *^*IL2rg*^*tmWjl*^/Sz (NOD/SCID/IL-2Rγ^null ^or NSG), which can be reconstituted with a human hematopoietic system through engraftment of human cord blood CD34^+ ^cells [9]. These offer unique opportunities to study human grade immunomodulatory reagents. The development of spontaneous tumor models in transgenic mice (in which animals are tolerant to genes used to induce the malignant event) offer multiple advantages over transplantable tumors for many applications. The tumors in models using genetically engineered mice (GEM) often develop similar defects in the tumor microenvironment, limiting host immune responses. Moreover, tumor growth is quite heterogenic mimicking human tumors. The heterogenic phenotype of most GEM models requires larger numbers of animals to be studied to assess significance of the intervention. Unfortunately, the cost of generating and maintaining transgenic colonies of GEM can be prohibitive for many investigators. In addition, these models are usually based on the tissue-specific expression of a strong driver oncogene, which may overwhelm the immune-surveillance and immune-editing steps of cancer development. One example of an alternative approach to integrate an oncogenic signal in tissue has been recently reported [10]. Hydrodynamic co-delivery of genes encoding β-catenin (CAT) and MET or AKT induced steatotic hepatocellular adenomas that transitioned to hepatocellular carcinomas (HCC) or led to rapid induction of HCC, respectively. This innovative approach overcomes many of the afore mentioned limitations by providing a rapid and relatively inexpensive method for generating spontaneous tumors in mice of a specific MHC background, in specific gene knock-out, transgenic, or aged mice. Together the preclinical models remain an important "proving ground" for some classes of immunotherapies and for the evaluation of possible synergies with combination immunotherapies. While imperfect, advanced and spontaneous tumor models are still considered to be more useful than in vitro studies at informing clinical trial designs of novel agents and combination immunotherapy.

With regard to predicting safety of novel antigen-based cancer immunotherapies by using animal models, numerous limitations exist. Vaccination with antigens relies on the species- (and allele-) specific binding of antigen to human leukocyte antigen (HLA) receptors (in the case of short peptide antigens) and species-specific processing of antigens by a complicated interplay involving different proteasome species, other proteases, heat shock proteins, TAP transporter and finally, again, binding to HLA receptor (in the case of protein, long peptide, RNA or DNA vaccines). Even if mice were generated that expressed the appropriate HLA type and the human antigen sequences, such models might not adequately predict safety or autoimmune effects based on the diversity of the other components of antigen processing machinery involved.

Preclinical animal studies have also been used to assess potential toxicity of immunologically active agents. In the absence of in vivo preclinical data, in-vitro assays have been used to identify the 'minimum anticipated biological effect level' (MABEL). A recent report offers a protocol that provides increased sensitivity to detect soluble T cell stimulants [11]. Alternatively, micro dosing or flat dose escalation studies have been proposed. The lethal toxicity associated with chimeric antigen receptor (CAR) gene-modified T cells is an example where a preclinical model did not exist to appropriately test the potential toxicity [12,13]. The two reported cases led to both National Institutes of Health (NIH) and the US Food and Drug Administration (FDA) review and resulted in modifications to clinical trial design where the dose of adoptively transferred gene-modified T cells is escalated from a much lower dose than where toxicity was observed. As new agents and combinations of immunotherapies are evaluated, flexibility of the regulatory agency providing oversight will be critical for the efficient translation of these strategies to patients.

#### Opportunities

Could standards be suggested for investigators using preclinical models to improve the utility or interpretation of animal studies? Are there other instances when proof-of-concept studies in animals can be waived? Additionally, the limitation of assessing toxicity of immunological agents, specifically monoclonal antibodies, in non-human primates has been raised at several SITC conferences. These studies, due to their high cost, limit the number of agents that are moved to the clinic. How often are such studies instructive of clinical toxicities and when is it appropriate to discuss with regulatory agencies the elimination of these studies?

### 2. Delayed Institutional, Administrative and Regulatory Approval

The time to obtain approval to initiate a clinical trial has been identified as a critical hurdle for some investigators. In the global science community there are academic institutions where administrative review can add as much as seven months to the approval process. At other centers, thanks in part to standardized procedures and protocols, and institutional familiarity with the proposed investigational strategies, administrative and institutional review board (IRB) approval can be obtained relatively quickly. Consistent with the difficulties perceived in the U.S. to open trials, there has been a large movement of cancer trials to Europe and Asia due to the slow activation of trials in the U.S.

With regards to regulatory approval within the US, FDA reviewers must respond to the application for an investigational new drug (IND) within 30 days of submission. While this efficient review process provides no guarantee for rapid approval, the feedback that the agency provides, sometimes prior to the 30 day window, allows for modifications that can sometimes resolve issues and avert a clinical hold on the application. Health Canada employs the same 30 day rule for review of clinical trials. Similarly, the European Medicines Agency (EMA) has the option of an accelerated review procedure for products of major therapeutic interest. In contrast, regulatory agencies in some countries may take a year or more to approve a comparable application.

Another major difference between nations is the disparity in production requirements for the biologics or drugs used in the clinical trials. In the US, FDA exempts most Phase 1 drugs, including biologics, to adhere to Current Good Manufacturing Practice (cGMP) regulations [14]. In contrast, the European Union has implemented a rule that all early phase studies must be performed under GMP. While the use of GMP in the European Union is thought to have increased the quality of clinical trials, especially of investigator-initiated trials, it has clearly added significant cost and limited the capacity of many academic institutions to perform translational cancer immunotherapy trials.

#### Opportunities

A cost-benefit analysis of restrictions that limit translation of novel therapies to patients with advanced cancer may be appropriate. Are there other processes, short of GMP, that might be employed to increase quality but not the cost of some early phase clinical trials? This is a particularly important issue since there is great variability in access to facilities that function using cGMP and GMP guidelines that also have the technologies available to produce novel biologics developed by academia. Even when a facility can be identified, traditional funding mechanisms rarely pay for the production of the new biologic.

### 3. Complexity of Cancer, Tumor Heterogeneity and Immune Escape

Clearly cancer is a complex problem and this complexity has been identified as a critical hurdle to the application of cancer immunotherapy. The heterogeneity of the cells making up the cancer and their propensity to develop resistance to any form of therapy is well established [15,16]. Further, histology results suggest that a specific cancer, for example melanoma, is not a single disease, but likely 13 or more different diseases [17], all of which may ultimately be found to respond uniquely to therapeutic interventions [18]. Also, local stromal non-cancer cells have a direct influence on tumor progression and outcome [19], illustrating the complexity of tumor microenvironment. In addition to the potential heterogeneity within each tumor is the likelihood that tumor at each metastatic site is heterogeneous in expression of antigens, or lack thereof, and/or escape mechanisms; substantially increasing the complexity of the disease in each patient far beyond the simple categorization of that disease.

On top of the complexities directly related to the tumor are variables that can influence a patient's ability to generate and maintain an effective antitumor immune response. A major factor in this setting is the overall immune status of the patient. This is influenced by age, previous therapeutic interventions as well as by elements directly and/or indirectly related to the tumor. The status of the patient's immune system and its impact on clinical outcome has important implications for the identification of host-related prognostic markers, of host-related predictive markers to classical chemotherapies and radiotherapies as well as that of novel innovative immunotherapies. Unfortunately, there is no consensus on a biomarker(s) for assessing immune status of individuals enrolling in immunotherapy trials [20], however this should not prevent investigators from incorporating novel strategies to assess immune competence of patients enrolling in trials. Recent reports suggest that the immune signature at the tumor site, characterized by genetic or histological assessment, may predict responsiveness to therapy [21,4]. Additional studies have also shown that pre-surgical clinical trials can be used as a mode of investigating the impact of immunotherapeutic agents on human immune responses in both the systemic circulation and tumor microenvironment, thus providing a feasible platform on which to obtain crucial data that can then be applied to larger clinical trials [22,23]. Support for these types of Phase Ia or Phase IIa trials [24], which are designed to investigate mechanisms and biologic endpoints, is necessary in order to identify potential biomarkers that correlate with benefit or resistance to therapy.

While additional validation is required, these observations are encouraging investigators to redouble their efforts to assess immune competence of patients entering immunotherapy trials. Also important to these efforts, is the need to encourage testing of new agents in the neo-adjuvant setting to allow improved assessment of potential biomarkers of early response.

Another level of complexity is the ability of cancer cells, under the selective pressure of an antitumor immune response, to shed targets or accessory molecules in ways that allow them to evade detection and killing by immune cells [25-27]. Alternatively, tumors may express inhibitory molecules that impair the antitumor immune response and limit the impact of the therapeutic intervention. While the complexity of this problem is considered a critical hurdle, appreciating this complexity and designing therapeutic combinations to augment immune responses and neutralize escape mechanisms holds substantial promise for improving the effectiveness of cancer immunotherapy.

#### Opportunities

Since the characterization of tumors prior to and following immunotherapy has not been well studied, the consortium might encourage a multicenter evaluation of such specimens. This could include the development of a taskforce to provide input on a global standardization of the tumor microenvironment. In support of this concept on October 24-25, 2012, SITC will provide opportunities for the consortium to gather in North Bethesda for a two-day workshop on evaluation of the tumor microenvironment. Performing systematic biopsies of tumor lesions considered as representative targets should also be considered and ethically admitted in most protocols to allow a dynamic characterization of immunomodulation. Further, modifications to some informed consent documents should be considered to ensure that patient specimens could be used to aid biomarker development. Additionally, better identification of major immune defects in patient groups may lead to more appropriate therapies.

### 4. Limited Availability of Reagents for Combination Immunotherapy Studies

While many preclinical studies have documented significant synergies and improved outcomes when immunotherapy is combined with a wide range of agents, trials with combined agents may present additional complexities and risks to the drug developer and patient. One problem is the classical method to find the maximum tolerated dose (MTD) in phase I studies. Biological products, in particular vaccines, have less toxicity and may have a bell-shaped dose immune response curve. This has promoted the idea of dosing based on biological activity assessed by a biomarker.

#### Opportunities

Developing a strategy that takes into consideration both toxicity grade and the "immune response score" could provide an optimal biologically active dose. While some investigators are implementing such strategies into their studies, consensus on this matter would likely aid the implementation of combination immunotherapy trials.

It is becoming increasingly apparent that many standard cancer treatments may enhance the effectiveness of immunotherapy, possibly due to increased inflammation, release of antigen and danger signals, immunogenic cell death pathways and dampening the effects of regulatory cells. Indeed, many investigators are exploring immunotherapy combinations with other immunotherapeutic agents, biologicals, targeted therapeutics, chemotherapy, radiation and/or surgery as promising strategies to improve cancer outcomes [28-32]. This enthusiasm has been driven by the appreciation that even agents long thought to work solely on tumor cells can have potent effects on the anti-cancer immune response.

For agents that are already approved, the hurdle may simply be limited resources or high costs necessary to acquire the specified treatment for a combination study unless the company marketing the product is willing to supply the agent for the study. However, for agents that are in early/late phase clinical trials and are not already approved, pharmaceutical sponsors may not want the added risk that the combination trial may interfere with their drug development and registration plan. One concern is that a novel strategy employing company A's agent X in combination with company B's agent Y, may result in a severe adverse event (SAE) that raises regulatory concerns about either drug, X or Y, as a single regimen. This may prompt additional patient safety monitoring requirements in all ongoing trials with drug X or Y, which pose particular challenges if either drug is in large, multi-national registration trials. Given the SAEs that have been observed with single agents (IL-2, anti-CTLA-4) and the limited experience with combining immune-potentiating biologicals, [33-35] there exists the possibility that combinations may increase toxicity. However, the potential to improve efficacy significantly, without concomitantly increasing toxicity, as has been observed in preclinical and a few clinical studies, provides a compelling rationale for combining immune-potentiating agents. It is important to continue the discussions in this area and try to agree upon a compromise that will allow earlier testing of combinations particularly in diseases that are in desperate need of new therapies. Most cancers are not cured by one agent. It is critical to take this into account and to work toward developing a mechanism for testing combinations where the scientific rationale supports the trial design.

Other concerns surround the possibility that investigators could discover something that might limit the utility of that drug or obtain negative results that devalue intellectual property (IP). Alternatively, mechanism of action studies may lead to broad claims by the investigators, further limiting a company's IP. Finally, integration of clinical and regulatory operational efforts between two companies poses challenges. These include selection of only one of the companies or academic institutions to hold the IND and assume full regulatory responsibility for a combination trial as well as dissemination of all single agent IND safety reports from each company to all investigators involved in the combination trial. If these hurdles cannot be addressed, it will take much longer to put together the "dream teams" of immunological agents that many in our field are eager to evaluate in the clinical setting based on synergisms observed in preclinical studies. At the 2010 Collaboration Summit on cancer immunotherapies hosted by SITC the ten participating organizations agreed that promoting innovative trials of combinations is a high priority. Late last year the NCI took constructive action by launching the Cancer Immunotherapy Network (CITN), providing a mandate to develop and conduct clinical trials with prioritized immunotherapy agents alone or in rational combinations [36-38]. While resources will be limited, the CITN establishes a cooperative, multicenter framework to advance a number of critical studies. But this is not enough. More needs to be done to enable exploratory trials of immunotherapy combinations.

#### Opportunities

One strategy may be to increase the number of academic manufacturing facilities that could provide clinical grade materials for clinical trials. Particularly for clinical grade agents that large pharmaceutical companies are not interested in and that small biotech may not be able to distribute to all the potential partners involved. This may be particularly helpful for vaccine components such as recombinant proteins, synthetic peptides, TLR agonists, etc. One solution would be to have GMP facilities supported in academic institutions, for instance in the pharmacy departments or faculties in universities or medical centers. Another option would provide government contracts to commercial laboratories to produce such products. Finally, governments might encourage corporations to more actively pursue these strategies by offering patent extensions or other incentives.

Recognizing the importance of promoting investigations of immunotherapy combinations, in March 2011 the CIC hosted its Annual Meeting with Focus on Schedule and Dose for Combination Therapies and in April, the CCIC also reviewed aspects of combination immunotherapy at their 4^th ^annual meeting. Additional meetings were held throughout 2011 with a focus on ways to improve immunotherapy outcomes. In May, CIMT met in Mainz, Germany, for their 9^th ^Annual meeting entitled "Targeting Cancer: Road-Maps for Success". From June 30^th ^until July 1^st^, The JACI met in Osaka for a symposium on the "Current status and future prospective of cancer immunotherapy". In September, CSCO and SITC hosted a joint cancer immunotherapy session in Xiamen, China, and in October, TIBT met in Jinan, China for their "12^th ^National Tumor Biotherapy Conference" and ESCII and NIBIT joined together in Siena for "New Perspectives in the Immunotherapy of Cancer". Also in October, the PIVAC held their 11^th ^meeting on cancer vaccines in Copenhagen. In November, the SITC hosted their second workshop on the science and logistics of combination therapy [39] and in December, SITC joined with NIBIT and the Italian Melanoma Intergroup in sponsoring "Melanoma research: a bridge from Naples to the World". In 2012 additional meetings focused on cancer immunotherapy are planned. In March the BDA will host their 11^th ^Biological Therapy of Cancer Conference in Munich and TVACT will host their 18^th ^annual meeting on Cancer Immunotherapy in Chicago. In April the CIC will host their annual colloquium outside Washington DC and in May CIMT will host their 10th annual meeting in Mainz. Early in 2012, the European Academy of Tumor Immunology will start writing combinatorial multicentric randomized Phase II trials associating academic GMP vaccines, immunogenic chemotherapy and immune checkpoint blockade inhibitors so that multiple institutions experienced in immunotherapy and immunomonitoring may be able to conduct this enterprise. While each organization will continue to pursue meetings and activities that address the needs of their members, the consortium of fifteen organizations, termed the World Immunotherapy Council, will work to find areas for collaboration and exchange of scientific information.

### 5. Limited Funds Available to Translate Science into Patients

Once investigators have identified a novel immunotherapy treatment, with compelling preclinical evidence to support its potential as a treatment for patients with cancer, the challenge of obtaining funding to initiate the clinical trial becomes a rate-limiting barrier. In the USA, reduction in funding by the National Cancer Institute (NCI) has seriously impacted the movement of new treatment strategies to the clinic. The Department of Defense has a number of programs that support translational clinical trials and this has helped fill the gap. The struggling biotech sector provides some help. In the USA some of this is through the NIH-funded Small Business Innovation Research (SBIR) and Small Business Technology Transfer (STTR) programs that have provided needed resources for moving agents to clinical trials. In other instances it is local and state governments, angel investors and philanthropy, more than high risk-adverse venture capital, that support these early phase trials. In the future it is expected that these sources will continue to play an important role in moving innovative first-in-human studies, particularly of cellular and combination immunotherapy studies, to patients with cancer. Investigators in Europe, Canada and Japan are also concerned about limited options to obtain support for translating new immunotherapy strategies to the clinic. However, the Japanese Ministry of Health, Labour and Welfare recently announced a fund of 1.1 billion Japanese yen for cancer vaccine clinical trials over the next 3 years. In China, the new 12^th ^5 year plan will provide broad support for translational clinical trials. Nonetheless, the majority of investigators and co-authors consider the difficulty in obtaining funding to initiate clinical trials to be a major hurdle for cancer immunotherapy.

#### Opportunities

To effectively communicate the impact investment in translational research and biotechnology/cancer immunotherapy has on the economic development of national and local economies as well as to human health [2].

### 6. Lack of Definitive Biomarkers of Immune Response

The lack of validated biomarkers for monitoring the development of an immune response following therapy is another critical hurdle for the translation of cancer immunotherapies. The iSBTc-SITC-/NCI/FDA Taskforce for Immunotherapy Biomarkers, composed of nine societies and participating organizations, has addressed this in detail [20,14]. Eight of the nine challenges identified by this Taskforce were related to immunological monitoring considerations. These included issues that should be optimized to obtain validated assays that can provide a reliable platform to compare cancer immunotherapy trials. A ninth challenge related to the identification of biomarkers for cellular immunotherapy products. These issues included:

1) Processing and storage of blood samples to bank peripheral blood mononuclear cells (PBMC) and serum for immunologic studies

2) Characterization of cellular products for therapy

3) Assay standardization and harmonization before testing patient samples

4) Centralization of immunological monitoring

5) Standardized assays that should be used for clinical trial antitumor immune response determination

6) How assay data should be analyzed for "responder" and "non-responder" identification

7) Reporting immunological monitoring data in publications

8) Validation of specific assays and/or analytes as biomarkers of clinical response

9) Novel assays in development for immunological testing of patients

Despite substantial efforts from many groups, immunological monitoring is challenged by two central limitations. First, we do not know which parameters of immune responses are the most important in a clinical response to immunotherapy; secondly, we do not know which assays or sample source (i.e., blood, lymph node, DTH site or tumor) are optimal to assess these parameters and correlate to efficacy. Indeed, the tumor-specific cellular immune response promoted by immunization often has not correlated with clinical cancer regression [40,41]. A contributing reason may be the inherent complexity of immune response assays, in conjunction with variable assay protocols across clinical trial laboratories, which results in high data variability and limited reproducibility [42]. Through more than five years of community-wide proficiency panels on the most commonly used immune response assays (ELISPOT, HLA-peptide multimers, ICS and CFSE) organized by the CIMT and CIC immune monitoring consortia, it could be demonstrated that assay harmonization is an effective mechanism to reduce these limitations [42-44]. Harmonization guidelines resulting from this process are simple to implement, do not impose standardized assay protocols on individual laboratories and improve assay performance without stifling scientific creativity. Assay harmonization may provide a solution for non-validated biomarker assays to minimize data variability and allow correlation of immune monitoring results with clinical outcomes [45].

Another major hurdle in biomarker identification is the low clinical response rates that limit identification of correlates with response to immunotherapies. Indeed, when response rates to immunotherapy reach 50%, it has been possible to identify a significant correlation with objective clinical response in patients maintaining at least 5% tumor-specific T cells in their peripheral blood for at least two weeks [46]. Standardized immune monitoring of large multi-institution trials has recently allowed for statistically significant correlations of anti-tumor immunity and clinical outcome [47].

#### Opportunities

Moving forward, the hurdles specified above will need to be addressed. The report from the iSBTc-SITC/FDA/NCI Taskforce on Immunotherapy Biomarkers [20] builds on the NCI's REMARK criteria [48] as well as other more recent reports, e.g., MIFlowCyt, MIACA, and MIATA [49-51]. Integration of standardized procedures and internal controls as well as improved reporting practices will improve the ability to identify immune biomarkers following immunotherapy and other approaches which impact immunity. The group will continue to promote discussion around the importance of standardization and support educational programs aimed at improving the ability to reproducibly assess immunotherapy biomarkers.

### 7. Conventional Response Criteria May Not Reflect the Patterns of Response to Immunotherapies

RECIST or modified WHO criteria have provided the basis for evaluating whether patients with cancer respond to therapy. These traditional criteria were developed for cytotoxic therapies and evaluate reduction in tumor burden following initiation of treatment. While immune therapies have led to striking and rapid reductions in tumor burdens in some patients, others have experienced progression prior to experiencing tumor regression or have had stabilization of disease. In these latter two instances, patients may ultimately recognize a benefit in overall survival but not be identified as responding to therapy based on conventional response criteria. This pattern of response to therapy has been observed by many investigators but was not systematically captured due to absence of adequate response criteria. In 2004, as part of a collaboration between the iSBTc (now SITC) and the CVC (now CIC) to address issues relevant to the development of cancer immunotherapy, both organizations formed the Cancer Vaccine Clinical Trial Working Group (CVCTWG), which included participation from the FDA and NCI. CVCTWG held several workshops between 2004 and 2005 with a concluding workshop jointly hosted by CVC and SITC at the 2005 Annual Meetings of both organizations. (http://www.sitcancer.org/meetings/am05/workshop.php). These workshops and the resulting publication with input from more than 180 investigators representing academia, NCI, FDA, and the biotech and pharmaceutical sector, discussed how evaluation of a clinical response to immunotherapy might be modified from that for cytotoxic agents [52].

Following the 2005 meeting, both collaborative and independent efforts of the CIC, CIMT and SITC took place to continue addressing these issues. Involvement from the NCI and FDA was included in many of these discussions. The goal of these meetings was to: a) summarize community knowledge, b) define challenges, and c) offer directions for improvement through community workshops. Resulting knowledge was used to systematically generate and analyze data to arrive at pertinent improvements of conventional clinical endpoints. Four main areas were addressed: 1) CIC and CIMT-CIP immune monitoring proficiency panels including >80 international laboratories across the field defined harmonization criteria to provide quality-control mechanisms and minimize data variability without standardizing laboratory protocols with the ultimate aim to allow for correlation with clinical endpoints [42,51,44]. 2) The SITC-FDA Taskforce on Immunotherapy Biomarkers, with input from 9 organizations, addressed the lack of validated biomarkers for monitoring the development of an immune response following therapy and identified 9 challenges critical for the translation of cancer immunotherapies [20] (see section "Lack of Definitive Biomarkers of Immune Response". 3) Clinical patterns of antitumor response for immunotherapeutic agents are more complex than those of chemotherapy [52-55] and adjustments to RECIST or WHO criteria to capture all patterns should be considered. 4) The translation of an immune response into clinical antitumor activity and possible survival benefit takes time [56,53,54]. Therefore, effects on patient survival may only be detectable several months after treatment start, which may be reflected in a delayed separation of Kaplan Meier curves. This observation was made as part of a systematic review of publicly available Phase 3 data from cancer immunotherapy trials during a CVC workshop in 2006 [56]. The delayed separation of Kaplan-Meier survival curves may be addressed through revised statistical methods of non-proportional hazards [54,57].

The core aspects of these community recommendations were reviewed at a United States Food and Drug Administration Workshop, which included participation and presentations by both CIC and SITC representatives, and were included in a draft guidance document on "Clinical Considerations for Therapeutic Cancer Vaccines" [58]. This illustrates how the collaborative efforts of community-based organizations can lead to an expansion of immunotherapy clinical trials methodology supporting further advances in the field.

#### Opportunities

The discussion on changes to response criteria needs to continue. A recent report used patient outcomes following treatment with ipilimumab, a monoclonal antibody that blocks CTLA-4, to evaluate how proposed new immune-related response criteria (irRC) compared to RECIST or WHO criteria [55]. The important observations from that report were that four patterns of response were all associated with favorable survival.

The four patterns of response to immunotherapy were:

1) shrinkage in baseline lesions, without new lesions;

2) durable stable disease (in some patients followed by a slow, steady decline in total tumor burden);

3) response after an increase in total tumor burden; and

4) response in the presence of new lesions.

The conventional response criteria assumed that early increase in tumor growth and/or development of new lesions indicated progressive disease, which has become synonymous with drug failure. For immunotherapeutic agents, however, initial tumor growth or appearance of new tumors does not necessarily reflect immunotherapy failure nor long-term outcomes and survival. The new irRC more accurately reflect the response patterns associated with immunotherapies, and may permit more comprehensive assessment of cancer immunotherapy clinical trial results as well as provide guidance in the clinical care of patients with cancer receiving immunotherapies. While these new irRC appear promising, prospective evaluation of these criteria following treatment with immune therapy is clearly warranted [57].

The FDA, who actively participated in many of these discussions, agreed that cancer vaccines might require considerable time in order to induce a therapeutic response. To address this the FDA provided specific recommendations for the clinical trial statistical analysis plan in their "Draft Guidance for Therapeutic Cancer Vaccines" [58]. It is important to note that the impact on survival is still the gold standard employed by the US FDA and that is the basis for the recent approval of sipuleucel-T and Yervoy [44,59]. While recent reports of markers of an immune response correlating with outcomes are encouraging, substantial opportunities remain for the development of novel surrogate markers of anti-cancer immunity that correlate with improved survival [47,60].

### 8. Paucity of Translational Teams of Scientists and Clinicians

While there are centers of excellence with teams of investigators working to translate the latest technologies, there are far too few for the number of diseases that need to be targeted with promising immunotherapies. This needs to be improved. Given the cost for drug development, industry alone cannot be relied upon to conduct all the early stage testing, particularly since academic translational investigator teams, close to both basic and clinical science, are likely in the best position to move "their" agent into the clinic. This requires an investment in infrastructure. Depending on the class of agent(s) and international setting, this may require simple clean rooms or a complete GMP facility. The necessary infrastructure, however, is not simply bricks and mortar, but human capital as well. Teams including regulatory staff for the substantial protocol and consent development and approval steps, QA/QC support, trained data managers and research nurses, in addition to clinicians and scientists, are required to make this work. Clinicians must be appropriately recognized for the time and energy they spend participating in clinical trials beyond their standard clinical duties (which are often more profitable). A common sentiment is that there is a dramatic shortage of clinicians with a commitment to clinical research. This may be due to health systems that poorly valorize involvement of clinicians in research. Another reason clinicians may not have developed a career path in immunotherapy may be linked to the previous negative experience of cancer immunotherapy. Perhaps the increasing momentum in the field will spark enthusiasm for clinicians to train in this field. Another limitation is the number of PhD scientists that are trained and empowered to move their science to the clinic. Recognition of this, particularly by the Howard Hughes Medical Institute (Med into Grad Initiative) and centers with NIH Clinical and Translational Science Awards (CTSA) has led to development of programs that are successfully targeting incoming PhD students in hopes of developing translational investigators [61]. But having clinical researchers and translational PhD scientists alone is not sufficient. The ability to organize, lead, motivate, meld and sustain multidisciplinary groups of investigator in translational teams is considered a critical hurdle for advancing cancer immunotherapies and has been recently discussed [62]. Recognizing the essential role that team science plays in translational cancer immunotherapy, the SITC, in celebration of their 25^th ^anniversary, developed an award to recognize centers that have excelled in this area and provided a significant and sustained contribution over the past 25 years [63]. Another signatory organization for this document, the Cancer Research Institute, has been a sustaining source of support for the field of cancer immunology for close to 60 years. Its Pre-doctoral and Post-doctoral Fellowship Programs have trained thousands of immunologists over multiple generations. More recently through its partnership with the Ludwig Institute for Cancer Research, its Cancer Vaccine Collaborative establishes the needed infrastructure, reagent procurement, clinical trials management, and funding to carry out coordinated early-phase clinical trials aimed at developing therapeutic cancer vaccines.

#### Opportunities

While the programs noted above provide a basis for training and supporting team science, the majority of Universities do not consider seriously these contributions when evaluating candidates for promotion and tenure. Recognizing the contributions of teams to the advance of translational medicine and human health and developing a structure for evaluating these contributions is an opportunity for this consortium.

### 9. Need to Enhance Exchange of Information Critical to Advancing the Field

Another component of this "team" hurdle is the exchange of information. Given the increasing complexity it is becoming less feasible for a single group to have the detailed knowledge and resources to investigate, analyze, select and implement the best strategies to move forward in clinical trials for any given indication. A possible solution to this hurdle may be to link clusters of investigators with interest and experience with a given tumor type. The histocompatibility/HLA field might serve as an example for this concept. In that field, participants from around the world supplied reagents, ideas, practical work and shared projects to advance the whole field of transplantation. As a whole, these investigators made progress by helping the entire field through specific input of work and resources, driving significant advances over several decades. The success of these interactions (workshops, exchanges, central repositories) laid the foundation for bone marrow transplantation and organ transplantation (kidney, heart, liver, lung), all of which would not have been feasible through the efforts of a single individual or organization, or even one regional or national consortium.

#### Opportunities

The CITN may be able to promote a similar activity as it brings together multiple groups under the same umbrella. Similarly, societies, primarily those represented by co-authors of this publication, could also play a role in bringing together groups of like-minded investigators. Through its annual meeting, associated programs and other collaborative initiatives, the SITC is committed to facilitating the exchange of information and education among basic and translational researchers, clinicians, and young investigators to advance cancer immunotherapies. Importantly, SITC and the other signatory organizations have initiated a process to join together and develop collaborative projects to catalyze continued success in cancer immunotherapy worldwide. This group, tentatively designated the World Immunotherapy Council, will begin by approaching some of the hurdles addressed in this document, and also by organizing joint scientific meetings and sessions.

## Conclusion

The identification of nine critical hurdles (Table 1) is an important beginning for this group of collaborating organizations focused on cancer immunotherapy. In late 2010, representatives of ten organizations met in Washington D.C. to discuss the formation of international working groups that can make recommendations to address these hurdles, facilitate change and improve the translation of novel immunotherapies to patients with cancer. Through this international, collaborative approach--marked by the establishment of the World Immunotherapy Council--the many investigators and the fifteen organizations involved in this initiative look forward to combining their efforts synergistically to accelerate the delivery of promising new cancer immunotherapies to patients around the world.

## Competing interests

BAF - Co-Founder UbiVac, SAB Micromet, SAB MannKind; PAA - participated in advisory board for Bristol Myers Squibb, GSK, Schering-Plough/Merck and Roche. He has received honoraria from Bristol Myers Squibb and Schering-Plough/Merck; NLB - employee of IRX Therapeutics, scientific advisor for Immunovaccine Technologies and Roche Canada, stock options for sanofi Aventis; CMB is an employee of Ribological GmbH; JAG - Employee of Alnylam Pharmaceuticals; KH - employee and stockholder of Celgene Corporation; RBH - Employee of Intrexon Corporation; AH - Employee Bristol-Myers Squibb; SJ - Founder and president of ZellNet Consulting; HIL - Employee of Roche; J-PM - Employee of Merck KGaA; HS-J - Co-founder and employee of Immatics Biotechnologies GmbH; WS - Employee of Takeda Pharmaceuticals; JMW - Employee Bristol-Myers Squibb; All other authors - No competing interests.

## Authors' contributions

BF prepared the manuscript collaboratively with input and review by all co-authors representing their respective organizations. All authors have read and approved the final manuscript.

## Consent

All individuals within the figures gave informed consent for publication of their image.
